# Mechanically facilitated micro-fluid mixing in the organ of Corti

**DOI:** 10.1038/s41598-020-71380-5

**Published:** 2020-09-09

**Authors:** Mohammad Shokrian, Catherine Knox, Douglas H. Kelley, Jong-Hoon Nam

**Affiliations:** 1 Department of Mechanical Engineering, University of Rochester, 203 Hopeman Engineering Bldg, Rochester, NY 14627 USA; 2 Department of Biomedical Engineering, University of Rochester, Rochester, NY USA; 3 Department of Neuroscience, University of Rochester, Rochester, NY USA

**Keywords:** Cochlea, Biological physics

## Abstract

The cochlea is filled with two lymphatic fluids. Homeostasis of the cochlear fluids is essential for healthy hearing. The sensory epithelium called the organ of Corti separates the two fluids. Corti fluid space, extracellular fluid space within the organ of Corti, looks like a slender micro-tube. Substantial potassium ions are constantly released into the Corti fluid by sensory receptor cells. Excess potassium ions in the Corti fluid are resorbed by supporting cells to maintain fluid homeostasis. Through computational simulations, we investigated fluid mixing within the Corti fluid space. Two assumptions were made: first, there exists a longitudinal gradient of potassium ion concentration; second, outer hair cell motility causes organ of Corti deformations that alter the cross-sectional area of the Corti fluid space. We hypothesized that mechanical agitations can accelerate longitudinal mixing of Corti fluid. Corti fluid motion was determined by solving the Navier–Stokes equations incorporating nonlinear advection term. Advection–diffusion equation determined the mixing dynamics. Simulating traveling boundary waves, we found that advection and diffusion caused comparable mixing when the wave amplitude and speed were 25 nm and 7 m/s, respectively. Higher-amplitude and faster waves caused stronger advection. When physiological traveling waves corresponding to 70 dB sound pressure level at 9 kHz were simulated, advection speed was as large as 1 mm/s in the region basal to the peak responding location. Such physiological agitation accelerated longitudinal mixing by more than an order of magnitude, compared to pure diffusion. Our results suggest that fluid motion due to outer hair cell motility can help maintain longitudinal homeostasis of the Corti fluid.

## Introduction

**Figure 1:**
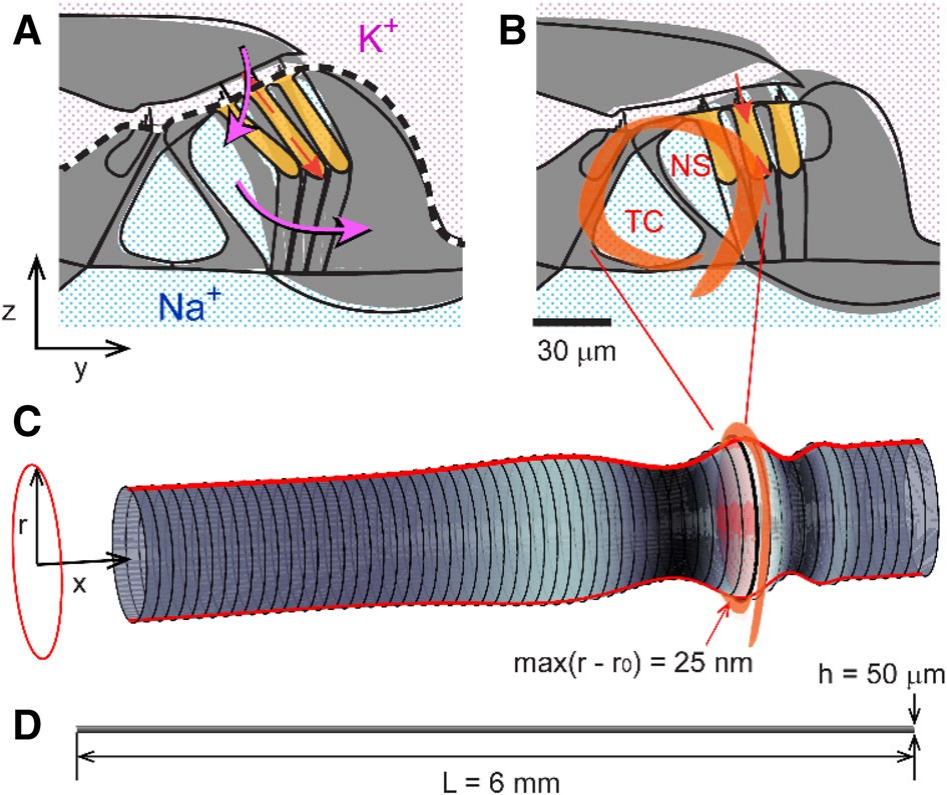
Corti fluid: extracellular fluid within the organ of Corti. (**A**) Mechano-transduction current (purple arrows) through the organ of Corti. The organ of Corti separates two fluids, rich in K^+^ and Na^+^, respectively. Cochlear mechano-transduction current is mostly carried by K^+^. Outer hair cells (orange colored) change their length due to the transduction current. (**B**) The subject of this study is the Corti fluid: the extracellular fluid within the organ of Corti including the tunnel of Corti (TC) and Nuel’s space (NS). As the organ of Corti expands (**A**) or contracts (**B**) due to outer hair cell motility, the Corti fluid moves. (**C**) Modelling the Corti fluid. The radial (y–z plane) section of the Corti fluid is simplified with a circle. Due to the traveling waves (red curves along the x-axis), longitudinal fluid motion occurs along the tunnel of Corti fluid. (**D**) A scale drawing illustrates the large aspect ratio of the Corti tube.

Acoustic pressure waves enter the cochlea at its base and propagate toward the apex, thereby causing the organ of Corti (OoC) to vibrate. The OoC, the auditory epithelium consisting of sensory receptor cells and their supporting cells, is sandwiched between acellular matrices called the tectorial and the basilar membranes (Fig. 1). The OoC vibrations are encoded into neural signals by a type of sensory receptor cells called the inner hair cells. The other sensory receptor cells, the outer hair cells, are cellular actuators that amplify small vibrations^1^. The top surface of the OoC separates two lymphatic fluids—K^+^-rich endolymph and Na^+^-rich perilymph (Fig. 1). There exists an electric potential, called the endocochlear potential, of approximately 80 mV between the two fluid-spaces. Both the chemical gradient and electric potential between the two fluid-spaces are generated and maintained by the stria vascularis^2^. Mechanotransduction current through the hair cells are mostly carried by K^+^ ions, and the current is driven by an electric potential—the difference between endocochlear potential and hair cell membrane potential. At rest (in silence), about half of transduction channels in the outer hair cells are open, causing the so-called silent current^3^. The silent current of outer hair cell is large for a cellular current—on the order of 1 nA.

The OoC has substantial extracellular fluid (Corti fluid), such as the fluid in the tunnel of Corti, Nuel’s space, and the outer tunnel (Fig. 1B). These extracellular fluid spaces in the OoC are inter-connected through the gaps between the outer hair cells, and pillar cells—the gaps are as large as a few micrometers^4^. For individual hair cells, an influx of K^+^ through mechanotransduction channels is balanced with efflux through basolateral K^+^ channels^3^. K^+^ extruded out of the outer hair cells is constantly resorbed by the spiral ligament fibrocytes and relayed to stria vascularis where K^+^ is secreted back to the endolymph space. How K^+^ is transported from outer hair cells to ligament fibrocytes remains unclear. There is evidence that K^+^ is diffused through the Corti fluid before being absorbed by the supporting cells^5,6^. The supporting cells in the OoC are connected by the gap junctions through which K^+^ can be transported^7^. Meanwhile, no gap junctions have been detected between hair cells and supporting cells. Due to the proximity to K^+^ source (hair cells), Corti fluid has higher K^+^ level than the perilymph in the scalar tympani (the cavity below the OoC)^6^.

Longitudinal inhomogeneity of the Corti fluid is the subject of this study. The OoC cross-sectional area becomes larger toward the apex of cochlea (> a factor of 2 in the gerbil cochlea^8^), while the transduction conductance becomes greater toward the base (> a factor of 5 in the gerbil cochlea^9^). This implies that there is more than ten times of burden to clear K^+^ in the Corti fluid toward the base of the cochlea. We explored if mechanical advection can alleviate the burden of cochlear fluid homeostasis in the basal cochlea by longitudinal fluid mixing. This hypothesis was motivated by two previous observations. A study reported longitudinal fluid motion along the tunnel of Corti, correlated with the outer hair cell motility^10^. Another study showed that the top and bottom surfaces of the OoC vibrate out-of-phase at stimulations below the best-frequency^11^, implying the cross-sectional area change of the OoC. The cross-sectional area change along the cochlear traveling waves is reminiscent of peristalsis. In this study, we use computational simulations to test the hypothesis.

Despite the previous observations, fluid mixing in the Corti fluid is non-trivial in terms of fluid physics. First, the Corti fluid space is too small in size. The Corti fluid space is a micro fluid tube of which dimensions are comparable to a human hair (Fig. 1D). Fluid mixing is commonly achieved by driving turbulence. However, turbulence is virtually impossible in the Corti fluid because of its small size. Second, OoC vibrations are infinitesimal. There are examples of mass transport and fluid mixing in biological tubes, such as fluid flow in the urethra due to pulsating vessel walls^12^, and mixing in stomach^13^. In those cases, the vibration amplitude is comparable to the thickness of the tube. In contrast, the vibration amplitude of the OoC is two to three orders of magnitude smaller than its thickness. Moreover, the ends of the Corti tube is closed unlike those other biological tubes. We investigated fluid dynamics and mass transport within a micro-tube with similar dimensions as the Corti fluid tube, to examine whether advective flows can enhance fluid mixing.

## Methods

The subject of this study is the Corti fluid, extracellular fluid within the OoC (the circled part in Fig. 1B). The space it fills is a long, coiled tube penetrated by columnar structures such as pillar cells and outer hair cells. For simplicity, this space is represented with a straightened circular tube (Fig. 1C). After assuming axisymmetry, the model is further reduced to a two-dimensional rectangular box. Throughout this paper, plots are not to scale: aspect ratio and displacement are heavily exaggerated. Therefore, we draw readers’ attention to the scales of our subject before continuing. The length of the mammalian cochlear coil is on the order of millimeters (35 mm for human, 7 mm for mouse). The thickness of the OoC ranges 40–100 µm^8^. The vibration amplitude of the OoC ranges from less than a nanometer for soft sounds to hundreds of nanometers for loud sounds. Figure 1C illustrates a traveling wave whose envelope spans a few millimeters. In realistic scale (Fig. 1D), the tube of Corti fluid looks like a line.

The pressure (*p*) and velocity (**u**) of an incompressible fluid is described by the Navier–Stokes equations and continuity equation^14^.

1 $$\frac{{\partial {\mathbf{u}}}}{\partial t} + {\mathbf{u}} \cdot \nabla {\mathbf{u}} = - \frac{1}{\rho }\nabla p + \nu \nabla^{2} {\mathbf{u}},\;{\text{and}}$$

2 $$\nabla \cdot {\mathbf{u}} = 0,$$

where *ν* is the kinematic viscosity, *ρ* is the fluid density, and *t* is time. Fluid viscosity has been considered^15–19^ or neglected^20–23^ per the purpose of studies. To our knowledge, no previous study regarding cochlear fluids analyzed the effect of advection (the second term of Eq. 1), because its contribution is negligible in the view of cochlear mechanics, while its nonlinearity increases computational expense. However, both terms play important roles in the mixing processes that motivate this study, so we retain both, despite substantial computational expense. Without the advection term, fluid particles oscillate about stationary points for cyclic stimulations such as sounds. We examine whether drift due to advection can homogenize the Corti fluid more quickly.

**Figure 2:**
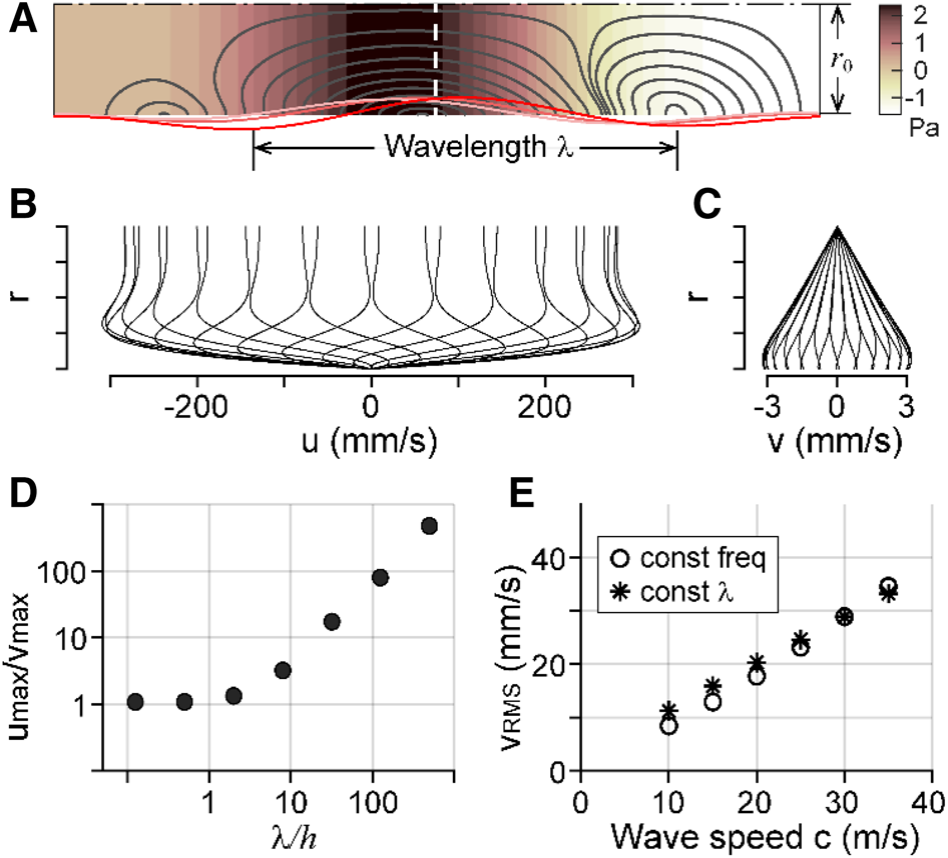
Fluid velocity driven by traveling waves. (**A**) The simulated fluid domain is long and narrow similar to the tunnel of Corti: 6 mm long and 25 μm thick. There is no net flux across any of four boundaries as if an isolated fluid domain. The bottom boundary is subjected to transverse vibrations in the form of longitudinal traveling waves of wavelength λ. The vibration amplitude was 50 nm where the spatial peak occurs at the center. The color contour and the curves indicate the pressure field and streamlines at a moment of time, respectively. (**B**, **C**) Longitudinal (u) and transverse (v) components of the fluid velocity at the midsection [the vertical broken line in (**A**)]. Curves indicate the velocity profile at different moments of an oscillatory cycle. (**D**) The ratio between velocity components depends on the wavelength. (**E**) Root-mean-square velocity of fluid (*v*_*RMS*_) is proportional to the speed of traveling wave propagation. Different wave speeds were given by varying either stimulating frequency (square) or wavelength (circle).

After approximating the Corti fluid space to a circular tube and assuming an axial symmetry, the geometry was reduced to a 2-D rectangle (Fig. 2A). The top boundary was considered the line of symmetry. The bottom boundary was subjected to vibrations, representing the cross-sectional area change of the fluid space. The left side was rigid, while the right side was open (pressure release). Before considering physiological vibrations, we investigated the effect of traveling wave parameters on fluid mixing using a simple dispersive wave. A wave with wavelength *λ* and frequency *f* was considered such as

3 $$r = r_{0} + a \sin \left( {\pi x/L} \right)\sin \left( {2\pi \left( {x/\lambda - ft} \right)} \right),$$

where *r* is the tube radius, *r*_0_ is its undeformed value, and *a* is the wave amplitude. The wave envelope of sin(*πx/L*) was to reduce boundary effects by decreasing wave amplitude towards the boundary (*x* = 0 or *L*). While the values of *f*, *λ*, and *a* were chosen within the physiologically reasonable range, the traveling wave created by Eq. 3 lacks features of natural cochlear traveling waves. For instance, in natural cochlea, the wavelength of traveling wave decreases as it propagates toward the apex until it becomes zero where the wave stalls. Likewise, phase velocity decreases as wave propagates. Initially, we neglected these complexities to investigate the effect of wave parameters on fluid mixing. A simulation of physiological traveling waves is presented later.

The governing equations were solved using COMSOL Multiphysics in a computer (Intel Xeon W-2145 processor). There were approximately 90,000 triangular elements as well as 8,000 quadrilateral elements in the boundary layer regions along the bottom and the left boundaries. Both of the element types used the third-order shape functions. Mesh size was 1–2 μm for the triangular elements, and it was reduced by a factor of four toward the boundaries, considering the boundary layer thicknesses estimated from the diffusive distance of an oscillating plate ($\delta \sim \sqrt{\nu /\omega }$). The resulting mesh could resolve the steep velocity gradient near the boundaries (Fig. 2B). The generalized-α method, a second-order implicit method^24^, was used for time-integration. A time-step size of 1/50th of the stimulation period was chosen.

Considering expected time scale for mixing (hours) and the time step size (µs), computing time was a practical challenge. We took advantage of the simulating condition—oscillatory vibrations. As the fluid flow under our considered condition is laminar, the flow pattern became cyclic after a brief transient period (< 1 ms). The convergence to a steady flow pattern was confirmed by comparing velocity fields after consecutive cycles. It took about 4 h to simulate 10 cycles, after which the cyclic solution reached a steady state (*corr*_*V*_ > 0.999, where *corr*_*V*_ is the correlation coefficient between the velocity fields at the completion of consecutive cycles).

The Navier–Stokes equations were solved, producing velocity and pressure values at fixed points in space. At any of those points, velocity and pressure oscillate periodically over a stimulating cycle. Due to the nonlinear advection term, however, fluid particles do not return to the same position after a cycle. Net displacement over a longer time scale than a cycle is known as the Stokes drift. The drift of particles over the time scale of diffusion (between minutes and hours) can be better represented by removing the oscillatory component of fluid particle motion. The trajectory ${\mathbf{x}}_{p}\left(t\right)$ of a fluid particle *p* can be calculated by integrating particle motion over a cycle:

4 $${\mathbf{x}}_{p} (t + T) = {\mathbf{x}}_{p} (t) + \int_{t}^{(t + T)} {{\mathbf{u}}_{{\text{E}}}({\mathbf{x}}_{p} ,\tau )d\tau} .$$

Here ${\mathbf{u}}_{\mathrm{E}}\left({\mathbf{x}}_{p},\tau \right)$ is the velocity experienced by the particle over time. Calculating the paths of many particles over a long time is computationally expensive, but since the flow is periodic, we need not integrate further than the period of stimulation *T* (= 1/*f*) as long as sufficiently many particles have been tracked. The trajectory of each particle can be extended by appending the one-period path that begins where the prior one ends. Repeating the procedure allows determining particle drifts over long times and significantly reduces computation expense. The Stokes drift velocity **u**_*D*_ is defined for the particle passing through location ${\mathbf{x}}_{p}$.

5 $${\mathbf{u}}_{D} \left( {{\mathbf{x}}_{p} \left( {t + 0.5T} \right),t + 0.5T} \right) = \left( {{\mathbf{x}}_{p} \left( {t + T} \right) - {\mathbf{x}}_{p} \left( t \right)} \right)/T.$$

The concentration *C* of interested ions evolves over time because of diffusion and advection. For the time scale of Corti fluid diffusion, the effective advection can be represented by the Stokes drift velocity, so that the dynamics is described by^14^

6 $$\frac{\partial C}{{\partial t}} = D{ }\nabla^{2} C - {\mathbf{u}}_{D} \cdot \nabla C,$$

where *D* is the diffusion coefficient of potassium ions in the Corti fluid. Since this is nonconservative form of advection–diffusion equation, this advection diffusion equation is independent of the Navier–Stokes equations. Thus the time and spatial grid sizes of the two governing equations are not the same. The time step size for Eq. 6 was initially 1 ms and adaptively increased over time. There were about 100,000 first-order triangular elements of which dimension was 1–2 μm. Numerical error estimated from mass conservation was < 10^–9^ per a stimulation cycle for Eqs. 1 and 2 and < 10^–4^ over a second for Eq. 6. The model parameters are summarized in Table 1.

**Table 1:** Model parameters.

Symbol (unit)	Value	Description
*L* (μm)	6,000	Tube length
*r*_0_ (μm)	25	Tube radius
*a* (μm)	0.025	Wave amplitude
*λ* (μm)	200–4,000	Wavelength
*c* (m/s)	1–100	Wave speed
*f* (kHz)	5–20	Frequency
*ρ* (ng/μm^3^)	0.001	Fluid density
*ν* (μm^2^/ms)	700	Kinematic viscosity
*D* (μm^2^/ms)	0.7	Diffusion coefficient

It should be noted that this study cannot discuss ion homeostasis because the source and the sink of ions are not incorporated. The mixing (homogenization) of fluid in micro-tube is the topic of this study. Given an assumed initial inhomogeneity, the time course of homogenization is simulated. To evaluate the mixing performance, in the following section, a mixing parameter is introduced. The parameter depends on the spatial distribution of ions, but not the absolute ion concentration.

## Results

### Longitudinal motion of Corti fluid

A 6 mm-long and 25 µm-thick fluid domain was subjected to traveling waves along one longitudinal boundary (Fig. 2A). For the case shown in panels (A–C), the wavelength was 4 mm and wave speed (phase velocity) was 80 m/s to the right (the longitudinal direction). The vibration amplitude was 25 nm. The color contour in panel A shows the pressure field when the peak inward (transverse) displacement occurred at the center of the tube. The streamlines (solid curves) depart from and arrive at the stimulating boundary. Velocity profiles at the midsection (vertical broken line in panel A) reveal that the flow moves back and forth in both directions as pressure waves propagate (panels B and C). The velocity in the longitudinal direction is greater than the transverse direction by two orders of magnitude for the simulated case. This implies that the fluid motion is primarily longitudinal. The longitudinal velocity that is set to zero at the stimulation boundary grows steeply over 5 µm to reach 300 mm/s. That is, approximately 75% of the fluid volume is under the influence of strong longitudinal motion. In contrast, the transverse velocity is greatest along the stimulating boundary and decreases toward the line of symmetry.

Long waves (*λ*/*r*_*0*_ >  > 1) cause large longitudinal motion, as shown in Fig. 2D. In contrast, as the wavelength gets shorter (*λ*/*r*_*0*_ < 1), longitudinal and transverse velocities become comparable. For a long wave, there was minimal pressure gradient in the transverse direction, thus the fluid was forced to move longitudinally. On the contrary, for a short wave (*λ*/*r*_*0*_ < 1), the pressure varied appreciably in both transverse and longitudinal directions (results not shown). Mammalian cochleae have *r*_*0*_ < 50 µm and experience wavelengths ranging from 100 µm to a few millimeters, so the long-wave case shown in panels A–C represents the natural conditions better than the short-wave cases.

Overall fluid velocity was proportional to the vibration amplitude *a* and the wave speed *c* (= *fλ*). As shown in panel E, as long as the wave velocity is the same, combinations of different frequencies and wavelengths resulted in comparable fluid velocity. In the plot, fluid velocity was represented by the root-mean-square velocity over the entire fluid domain.

### Fluid trajectories obtained from Stokes drift velocity

**Figure 3:**
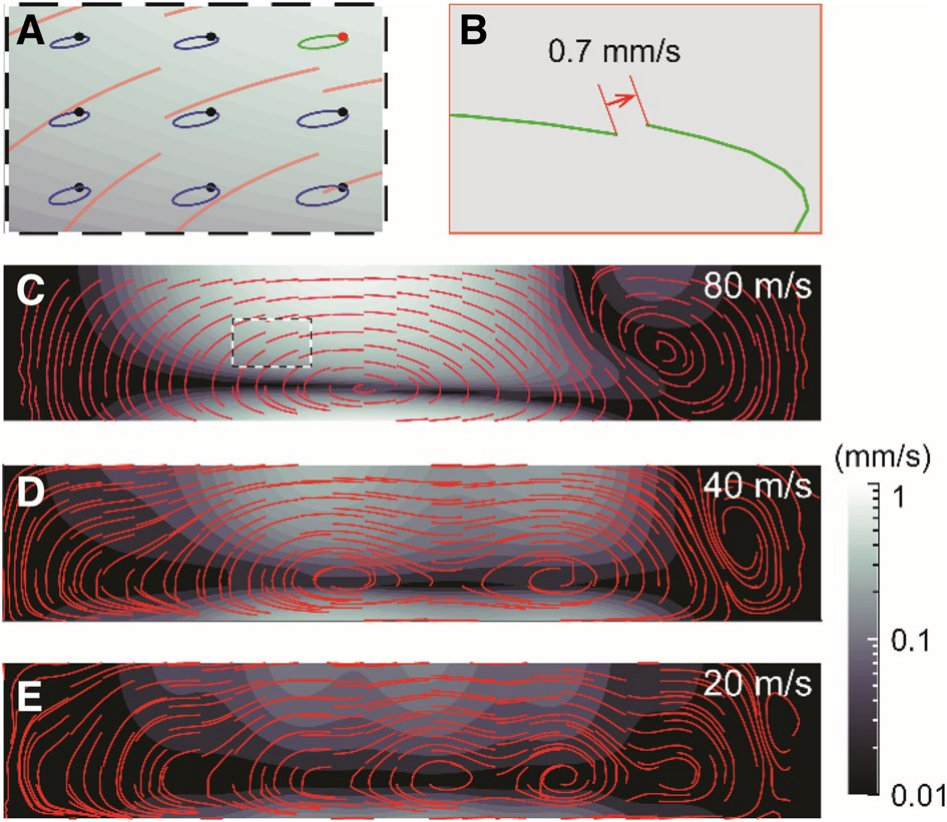
Advective fluid motion due to traveling waves. (**A**) Illustration of particle paths over a cycle of vibration (not to scale). (**B**) An enlargement of a particle motion [the green curve in panel (**A**)] shows that a particle does not return to its original position, resulting in a drift velocity of 0.7 mm/s (actual value). (**C**–**E**) Advection of fluid. The red curves are fluid particle trajectories due to Stokes drift. The color contour represents the amplitude of the Stokes drift velocity. The results of three wave-speeds are shown (c = 80, 40 and 20 m/s).

The result in Fig. 2 shows large instantaneous longitudinal fluid velocity when long waves propagate at high speed. However, this does not necessarily result in longitudinal mass transport because fluid particle motions are primarily cyclic—they return to nearly the same position after each cycle. Effective long-term mass transport is caused by the offsets particles experience over many cycles, due to Stokes drift. For the case of Fig. 2A–C, the velocity field was analyzed to quantify those offsets (Fig. 3).

Figure 3A shows the paths of nine particles over one cycle (the sub-domain of panel A corresponds to the broken rectangle in panel C). As expected, particles return to nearly the same position after a cycle but experience small offsets. Corresponding Stokes drift velocity was between 0.1 and 1 mm/s over half of the fluid domain in the case of c = 80 m/s (Fig. 3C). This Stokes drift velocity is smaller than the peak instantaneous velocity by more than two orders of magnitude (cf. ~ 300 mm/s in Fig. 2B). Integration of the Stokes drift velocity **u**_*D*_ results in the trajectories in Fig. 3C–E (red curves) that approximate fluid particle motions after excluding fast oscillatory motion. The oval shape of particle paths is aligned with their major axis parallel to the longitudinal direction, consistent with the velocity ratio between the two directions. Stokes drift is a nonlinear effect and cannot be observed if the advection term of Eq. 1 is neglected. The advection patterns along which the particles move are relevant to fluid mixing.

The Stokes drift velocity field that represents these vortex patterns is a way to better visualize these traveling paths for greater time scale than the period of vibration. The vortex patterns were affected by wavelength and boundary conditions. For example, the right-most vortex was generated due to the rigid boundary along the right edge. The other vortices have sizes roughly comparable to the wavelength (4, 2 and 1 mm for panels C, D and E, respectively). The direction of advective flow was counterclockwise—along the wave propagation direction near the boundary and against the wave propagation direction in the middle. As fluid particles tend to circulate within vortices, long vortices are expected to enhance longitudinal mixing more than short ones. These velocity fields shown in Fig. 3C–E were used as the advection velocity (**u**_*D*_) in solving Eq. 6.

### Advection and diffusion

To evaluate fluid mixing in terms of ion concentration, advection–diffusion equation (Eq. 6) was solved for different mechanical agitations. Diffusion in our study was nearly one-dimensional due to the geometry (i.e., the length dimension is dominant). The fluid homogenized in the radial direction within a fraction of second, while it took over an hour along the longitudinal direction. Considering this one-dimensional aspect, initial concentration was given so that it varied linearly along the length of fluid tube: $C\left(x,t=0\right)={C}_{L}+({C}_{R}-{C}_{L})x/L$, where ${C}_{L}$ and ${C}_{R}$ are the initial concentration values at the left and right ends. Given values were 5 and 1 mM for ${C}_{L}$ and ${C}_{R}$, respectively. It should be noted that our results are based on normalized quantities. Thus, the absolute values of concentration do not affect our result, but the longitudinal pattern of concentration gradient matters. As the spatial grid for mass transport (advection–diffusion) was different from that for fluid dynamics, velocity values at grid points were interpolated from the fluid dynamics solution.

Over time, the fluid was mixed and homogenized due to diffusion and advection. To quantify the level of homogeneity, the mixing parameter *χ* was defined using the standard deviation of ion concentration across the fluid space:

7 $$\chi \left( t \right) = 1 - {\text{stdev}}\left( {C\left( t \right)} \right)/{\text{stdev}}\left( {C\left( 0 \right)} \right).$$

**Figure 4:**
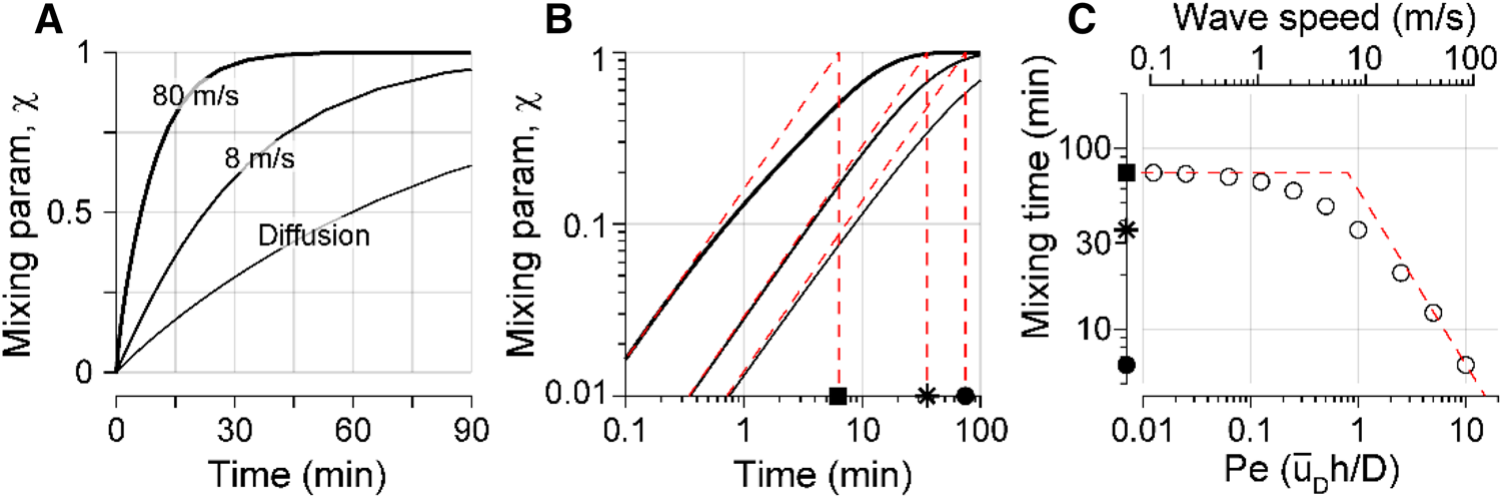
Mechanical agitation enhances fluid mixing in a micro-tube. (**A**) The mixing parameter χ (Eq. 7) increases over time from zero (initial status) to one (completely homogenized). The mechanically agitated cases (drift velocity fields from Figs. 2 and 3) are compared with the case of pure diffusion. (**B**) The same curves as in (**A**), but in the log–log scale. The effective ‘mixing time’ was defined as the intercept between the initial tangent line and χ = 1 (red broken lines). Mixing times corresponding to c = 80, 8 m/s and pure diffusion are indicated with different markers on the time-axis. (**C**) Mixing time decreased as wave-speed or Péclet number (*Pe*) increased. *Pe* is defined as ${\stackrel{-}{u}}_{D}h/D$, where ${\stackrel{-}{u}}_{D}$ is the root-mean-square drift velocity over the space. The broken lines are the asymptotes of the mixing time versus *Pe* curve.

The mixing parameter is zero initially and it approaches one as the fluid space becomes homogeneous. The absolute value of concentration does not affect the mixing parameter. Figure 4A shows the change of mixing parameter over time when the Corti fluid model is subjected to the mechanical agitation of Fig. 3C, and when there is no mechanical agitation (pure diffusion). When mechanically agitated, the fluid was homogenized quicker. To quantify the speed of homogenization, the mixing time was defined as the intercept between the initial tangent line to the curve of *χ*(*t*) and the line of *χ* = 1 (Fig. 4B). Initial slope was chosen to define the mixing time because the mixing at early stage characterizes the advective mixing well. In the mechanically agitated case, the mixing time was faster by up to one order of magnitude than pure diffusion (6.4 min for 80 m/s case versus 74 min for pure diffusion case, Fig. 4B). There existed a critical wave speed across which the dominant mode of mixing switched between diffusion and advection (Fig. 4C). For the considered micro tube and stimulation level, the critical wave speed was 7 m/s. The relationship between the mixing time and the wave speed is shown in Fig. 4C. The horizontal axis is also presented with the Péclet number (*Pe*), defined as $Pe={\stackrel{-}{u}}_{D}h/D$, where ${\stackrel{-}{u}}_{D}$ is root-mean-square Stokes drift velocity over the space, and *D* is the diffusion coefficient. The Péclet number represents the contribution in mass transport due to advection relative to diffusion. Consistent with the physical implication of *Pe*, the mixing time for advection–diffusion became inversely proportional to the wave speed when *Pe* >  > 1. When *Pe* <  < 1, the mixing time approached to the value of pure diffusion (filled square). Note that the physiological speed range (> a few m/s) corresponds to the domain of advective mixing.

### Physiological traveling waves and micro fluid mixing

**Figure 5:**
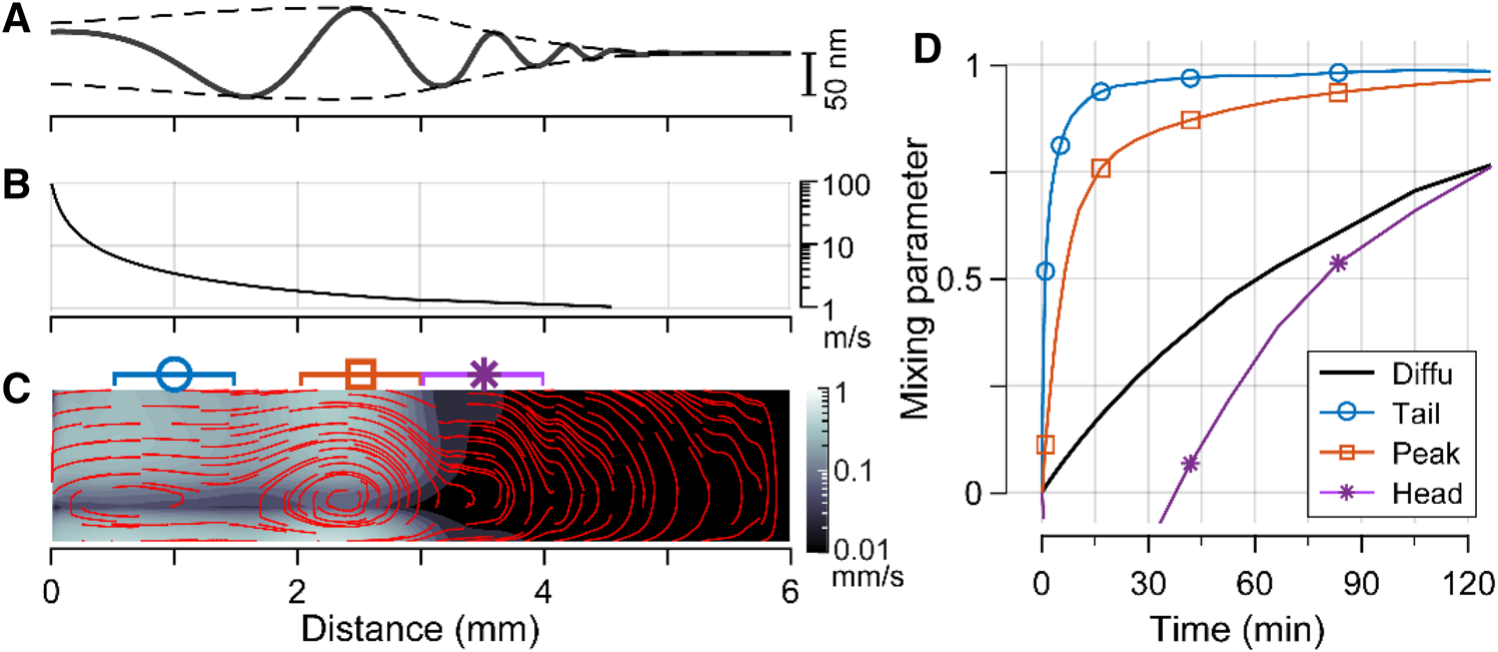
Advective mixing due to physiological traveling waves. (**A**) Physiological vibration pattern^25,26^. For such a pattern with 9 kHz at 70 dB pressure, peak vibration amplitude was 60 nm. (**B**) Wave speed (phase velocity) along the length. (**C**) Simulated advection of Corti fluid, represented as in Fig. 3. (**D**) Mixing of Corti fluid over time. Local mixing parameters of 1-mm span centered at 1, 2.5 and 3.5 mm were computed to represent mixing in the tail, peak and head region of cochlear traveling waves.

Physiological traveling waves differ from the simple traveling wave of Eq. 3 in that the wavelength gets shorter as the wave propagates toward the apex (Fig. 5A). As a consequence, the wave speed (phase velocity) decreases as the wave propagates (Fig. 5B). The wave pattern of Fig. 5A approximates a measurement of chinchilla cochlea^25^. The original measurement was made at a location where the best responding frequency was 9 kHz. Sound tones at different frequencies were delivered at 70 dB sound pressure level (SPL) and vibrations were measured at the basilar membrane (the bottom boundary of the OoC in Fig. 1A). From the frequency responses, the spatial vibration pattern was obtained using the scaling symmetry^26^. A rough approximation we made was that the measured basilar membrane displacement is comparable to the cross-sectional radius change (*r–r*_0_, in Eq. 3). Two recent observations may justify this approximation. First, there is significant phase difference between the top and bottom surfaces of the OoC (up to 180°^11^) which results in cross-sectional area change. Second, the top of the OoC vibrates more than the bottom (basilar membrane)^27,28^.

Using the same Corti fluid model as the one in Figs. 1, 2, 3 and 4, the stimulating boundary condition was replaced with this physiological traveling wave to obtain the results shown in Fig. 5. Stokes drift trajectories show fewer isolated vortices compared to the simple wave case in Fig. 3, despite multiple wave cycles over the simulated length. This resulted in fewer transport barriers that slow down longitudinal fluid mixing. The Stokes drift velocity sharply drops across the peak of wave envelope (x ≈ 3 mm, Fig. 5C). Instead of computing mixing parameter for the entire fluid domain, local mixing parameters in the tail (circle), peak (square), and head (rectangle) of the traveling wave were computed (Fig. 5D). The local mixing was considered for two reasons. First, we wondered in which region of traveling wave the fluid mixes better. As the wave parameters vary along the distance, the wave speed gets slower toward the apex, while the vibration amplitude peaks where the wave speed is slow (Fig. 5A,B). Second, cochlear fluid mixing must be a superposition of local events. Unlike the artificial case in Fig. 3, natural traveling waves do not fill the entire tube length—their wave envelopes have finite lengths. For instance, when the gerbil cochlea is subjected to a modest level of sound, 20 and 1 kHz sounds make approximately 2 and 9 mm-long wave envelopes, respectively^29^. The envelope length depends not only on frequency but also on sound pressure—louder sounds result in wider wave envelope. Waves with longer envelopes have higher mixing parameters if the entire domain is considered. Local mixing matters because, in reality, Corti fluid mixing is an ensemble of many waves. At a specific location, the most efficient wave component will dominate the mixing. Therefore, mixing of the entire cochlear length by a single wave does not represent Corti fluid mixing well.

The fluid mixing was more active toward the tail (*x* = 0) of the traveling wave. This location-dependence of mixing is reasonable considering that the wave speed decays (from 100 m/s to less than 1 m/s, Fig. 5B), while the vibration amplitude does not change dramatically between the tail and peak region. The local mixing parameter does not increase monotonically in the head region of traveling wave (indicated with rectangle, Fig. 5C,D); the mixing parameter decreased over the first 10 min.

## Discussion

### Taylor dispersion in the Corti fluid

The Corti tube seems like an unlikely place for vibration to enhance mass transport. Its small size makes turbulent mixing impossible (Fig. 1). Peristatic fluid transport from one end to the other seems unlikely because an end is closed (Fig. 2A). Furthermore, vibrations are infinitesimal—three orders of magnitude smaller than the OoC thickness for modest to loud sounds.

Despite such an adverse situation, we identified two conditions under which advection can enhance fluid mixing in the Corti fluid tube. First, the wavelength of cochlear traveling waves should be much longer than the OoC thickness (*λ*/*r*_0_ >  > 1, Fig. 2). Under this condition, the momentum of transverse vibrations effectively turns into longitudinal fluid motion. Second, the speed of wave propagation *c* should be greater than 7 m/s (Fig. 4). These two conditions are well satisfied by the mammalian cochlea. The OoC is 40–100 µm thick^8^, while the wavelength of physiological traveling waves is about 200 µm near the peak of the wave envelope and on the order of millimeters in the tail of traveling waves^30^. The speed of wave propagation (phase velocity) decreases as the waves propagate, dropping from over 100 m/s at the onset to a few m/s at the peak (Fig. 5B).

**Figure 6:**
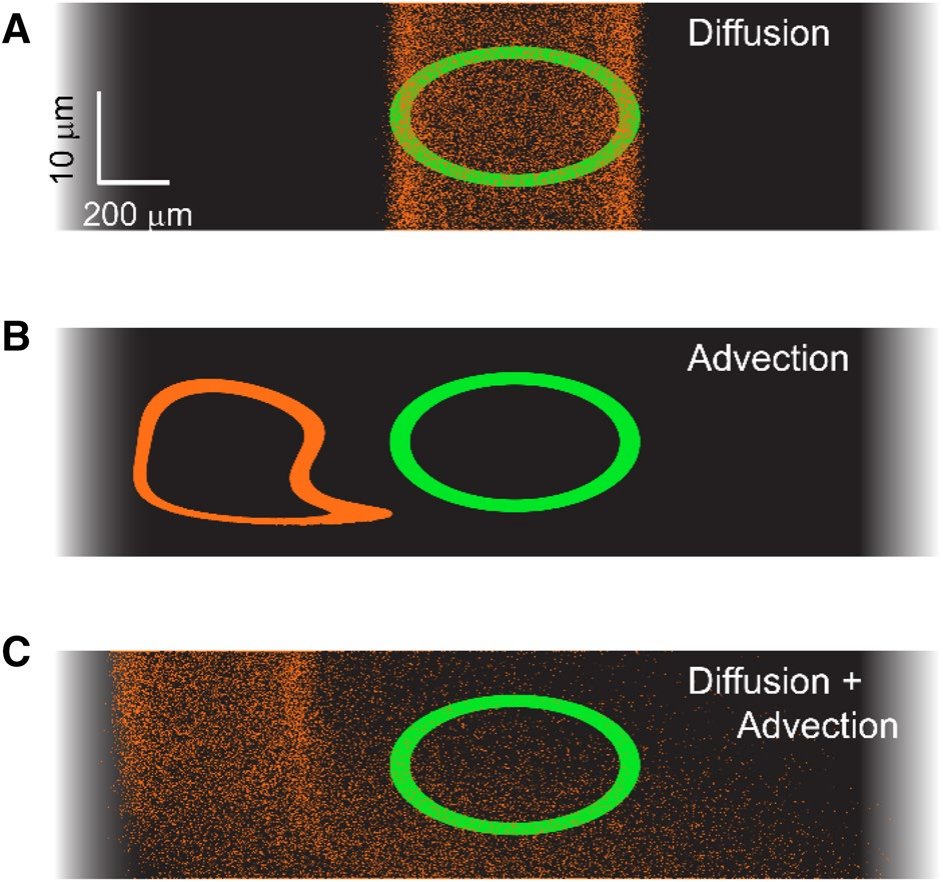
Mixing due to diffusion and advection. To visualize mixing, fluid particles along an elliptical ring were marked (green). The field of view spans 2.5 mm near the peak of traveling waves in Fig. 5. After 1,000 s, the particles drifted to new locations (orange). Three conditions were simulated: (**A**) pure diffusion without any advective flow; (**B**) pure advection when there is no diffusion (C_d_ = 0); (**C**) Realistic condition with fluid advection (Fig. 5C) and diffusion (D = 1 μm^2^/ms). The appearance that orange particles in panel (**A**) spread more in the vertical direction than in the horizontal direction is only an artifact of the exaggerated aspect ratio. An animated version of this figure is presented in Supporting Material.

The phenomenon we observe is consistent with the theory of Taylor dispersion^31^, in which mixing is enhanced because solutes diffuse across shear layers. To demonstrate the synergistic interaction between advection and diffusion, fluid particles in a simulation of the Corti fluid were tracked over time (Fig. 6). Initially, the particles were placed in an oval ring. Three conditions were simulated over 1,000 s. In one (Fig. 6A), there was no fluid flow, so the particles dispersed due to thermal random walks (diffusion). In another case (Fig. 6B), there was no diffusion (*D* = 0), but the fluid was subjected to the velocity field in Fig. 5C (near the peak of the traveling wave indicated with rectangle). The particles drifted due to the advective flow, but the mixing parameter hardly changed over the observed time span. In a third case, a realistic combination of advection and diffusion (Fig. 6C) facilitated fluid mixing.

### Considerations and consequences for natural hearing

Several simplifications were made for simulation that could affect mixing performance. First, the Corti tube was reduced to a circular tube and then to a two-dimensional rectangle after assuming axisymmetry for simplicity. As the fluid motion relevant to our conclusion is along the length, the assumption of axisymmetry may make minimal difference. Second, the Corti tube is not a uniform fluid space. It is penetrated by columnar microstructures such as the pillar cells and outer hair cells. They are likely to reduce mixing by slowing the flow but also enhance mixing by perturbing the flow pattern, like stirrers. Third, due to computational expense, we simulated pure tones. In natural hearing environments, the cochlea is subjected to numerous waves in which tail regions, where mixing is strongest, are spread all over the cochlear length. For this reason, our simulated condition might be conservative rather than exaggerated, and that mixing may occur more effectively under physiological conditions. Finally, the Corti fluid space was considered as if an isolated fluid domain. Unlike the top boundary of the organ of Corti tightly sealed with gap junctions, ions must diffuse across the basilar membrane. In a previous study, Johnston et al.^6^ observed an increase of K^+^ concentration as the probing electrode penetrated the basilar membrane toward the Corti fluid. This implies that the basilar membrane has a finite permeability for potassium ions. While the findings of our study hold regardless of basilar membrane permeability, if one wants to predict a specific potassium level, finite permeability of the basilar membrane will need to be considered.

It should be noted that, despite our result, higher sound intensity does not necessarily mean faster mixing. Our study suggests that the vibrations resulting in organ of Corti cross-sectional area change is the driving force for mixing. The cross-sectional area change might be caused by outer hair cell motility^11,32^ which is considered to saturate around 60 dB SPL. At a higher level, while mixing is not much better than 60 dB SPL case, potential damage due to excessive sounds may overwhelm the advantage of mixing. Our findings suggest that normal hearing environments with modest sounds are beneficial for hearing health. On the other hand, loss of outer hair cells or their motility will reduce the longitudinal fluid motion in the Corti tube and cause problems in fluid homeostasis in the organ of Corti. Although we focused on the specific example of potassium, our findings apply to any longitudinal chemical inhomogeneity. For instance, longitudinal advection could help clear away accumulations of metabolic waste.

Another example of longitudinal inhomogeneity in the cochlear fluid is found in cochlear drug delivery. The predictions of this study will be testable with cochlear drug delivery experiments. For instance, in clinical trials, drugs such as steroids and antibiotics are delivered locally though the round window because systemic delivery is considered inefficient due to blood-labyrinth barrier^33^. Typically, a solution with 100 times higher concentration than the half-maximal effective concentration (indicated with either EC_50_ or IC_50_) is applied at the round window so that the drug can diffuse across the window membrane and move along the scala tympani. There were experiments that measured the time of drug delivery along the cochlear length. In some studies, drugs affecting outer hair cell motility such as salicylic acid and gentamicin were used (e.g.,^33,34^). We predict that, when a drug not affecting outer hair cells is applied at the round window, the presence of sounds will facilitate drug delivery along the cochlear length.

## Supplementary information

Supplementary Information.
